# Cardiac Implications of Adenotonsillar Hypertrophy and Obstructive Sleep Apnea in Pediatric Patients: A Comprehensive Systematic Review

**DOI:** 10.3390/children11020208

**Published:** 2024-02-06

**Authors:** Marco Zaffanello, Refika Hamutcu Ersu, Luana Nosetti, Giulio Beretta, Massimo Agosti, Giorgio Piacentini

**Affiliations:** 1 Department of Surgery, Dentistry, Pediatrics, and Gynecology University of Verona, 37134 Verona, Italy; giorgio.piacentini@univr.it; 2 Division of Pediatric Respirology, Children’s Hospital of Eastern Ontario, University of Ottawa, Ottawa, ON K1N 6N5, Canada; rersu@cheo.on.ca; 3 Pediatric Sleep Disorders Center, Division of Pediatrics, “F. Del Ponte” Hospital, University of Insubria, 21100 Varese, Italy; luana.nosetti@uninsubria.it (L.N.); gberetta8@studenti.uninsubria.it (G.B.); 4 Department of Medicine and Surgery, University of Insubria, 21100 Varese, Italy; massimo.agosti@uninsubria.it

**Keywords:** A&T, adenotonsillectomy, ATH, adenotonsillar hypertrophy, OSA, obstructive sleep apnea, PH, pulmonary hypertension, SDB, sleep-disordered breathing

## Abstract

This review investigates the relationship between pediatric obstructive sleep apnea, often associated with adenotonsillar hypertrophy, and cardiovascular health, particularly pulmonary hypertension. We conducted a comprehensive literature search using electronic databases, including Medline Pub-Med, Scopus, and the Web of Science. The study analyzed a total of 230 articles and screened 48 articles, with 20 included in the final analysis, involving 2429 children. The PRISMA flowchart visually illustrates the selection process, and the ROBINS-E and –I tools help ensure the reliability and validity of the evidence produced by these studies. These studies explored various aspects, including the severity of obstructive sleep apnea, cardiac anomalies, cardiac stress markers, risk factors for pulmonary hypertension, and the impact of adenoidectomy and tonsillectomy on cardiac function. The research found that adenotonsillar hypertrophy and obstructive sleep apnea are significant risk factors for cardiovascular complications, especially pulmonary hypertension, in children. Adenoidectomy and tonsillectomy may provide effective treatments. Following adenoidectomy in relation to obstructive sleep apnea, there appears to be a reduction in mean pulmonary artery pressure during echocardiographic examination. However, the efficacy of these procedures can vary based on the severity of obstructive sleep apnea and individual cardiac conditions. The study also identified concerns regarding data bias. The authors emphasize the need for well-designed clinical studies, including both healthy patients with adenotonsillar hypertrophy and vulnerable children with genetic disorders, to ensure that clinical decisions are based on solid scientific evidence.

## 1. Introduction

Obstructive sleep apnea (OSA) in the pediatric population is a clinical condition characterized by complete or partial upper airway obstruction during sleep. Clinical history typically includes symptoms such as snoring, laboured breathing during sleep, daytime sleepiness, and learning or behavioural issues [1]. Epidemiological studies revealed that the prevalence of OSA in children ranges from 1% to 5%, solidifying its status as a relatively common condition among the pediatric population [2]. In individuals suffering from OSA, the normal airflow during sleep is significantly reduced or, in severe cases, wholly obstructed due to anatomical abnormalities in the upper airway, most notably adenotonsillar hypertrophy (ATH) in children [1]. OSA has been linked to disruptions in growth, neurobehavioral function, and inflammatory processes. Sleep-disordered breathing (SDB) in the pediatric population, including OSA, may be associated with cardiovascular complications [3].

The complex relationship between inflammation and OSA is marked by its intricacy and the potential for bi-directionality. In particular, OSA encompasses various facets of the inflammatory response. Notably, intermittent nocturnal hypoxia has been associated with increased oxidative stress, elevated pro-inflammatory markers, and decreased endothelial function [4]. Consequently, endothelial dysfunction is closely linked to cardiovascular events and the progression of cardiovascular diseases [5]. This multifaceted interplay underscores the necessity for a comprehensive understanding and management of OSA in pediatric patients. Children with OSA face an elevated risk of experiencing autonomic dysfunction, endothelial impairment, and ventricular re-modelling. Previous studies indicated that OSA substantially increases the risk of hypertension, arrhythmias, ventricular morphological abnormalities, ventricular contractility compromise, and right atrial pressure elevation in children and adolescents [6]. Risk factors for hypertension include older age, obesity, and the severity of OSA [7].

The prevalence of pediatric pulmonary hypertension (PH) is on the rise, owing to improved identification and increased patient survival, and it remains a substantial cause of morbidity and mortality [8,9]. The estimated incidence of sustained PH across all categories has been reported to range between 4 and 10 cases per million children annually [10]. Recent studies have advanced our understanding of pediatric PH, yet its management remains challenging due to the absence of evidence-based clinical trials [11]. Only a few studies have reported an association between OSA and PH in children and adolescents. Moreover, minor cohort studies showed improvements in left ventricular (LV) and right ventricular (RV) performance in young children and teenagers with moderate-to-severe OSA after adenoidectomy and tonsillectomy (A&T) [6].

Aims of the study:

The aim of this study was to examine the roles of ATH and obstructive sleep apnea (OSA) as risk factors in the development of various cardiac abnormalities. Additionally, the study aimed to assess the potential therapeutic value of A&T in improving cardiac function in children with OSA caused by ATH.

## 2. Materials and Methods

We searched the electronic databases of Medline PubMed Advanced Search Builder, Scopus, and Web of Science (WOS) using MeSH terms (https://meshb.nlm.nih.gov/, accessed on 31 August 2023) and the following text words:

WOS: TS = (“children” OR “infant” OR “pediatric” OR “Peadiatric”) AND TS = (“pulmonary hypertension” OR “ pulmonary artery pressure”) AND TS = (“sleep-disordered breathing” OR “sleep apnea”)

SCOPUS (EXPORT DATE: 31 August 2023): (“children” OR “infant” OR “pediatric” OR “Paediatric”) AND (“pulmonary hypertension” OR “pulmonary artery pressure”) AND (“sleep-disordered breathing” OR “sleep apnea”)

PUBMED (EXPORT DATE: 31 August 2023): (“children” OR “infant” OR “pediatric” OR “Paediatric”) AND (“pulmonary hypertension “ OR “pulmonary artery pressure”) AND (“sleep-disordered breathing” OR “sleep apnea”)

PICOS criteria:

The PICOS criteria [12] and key elements of the research for the selection of included studies were defined as follows:

Participants:

Inclusion: Children aged 1 to 18 years with a confirmed diagnosis of OSA, SDB, ATH, or adenoid hypertrophy, and children with snoring symptoms. All studies providing data on a significant number of participants (≥20). Children aged 1 to 18 years. Exclusion: Children with significant comorbidities or other medical conditions unrelated to ATH that could influence the analysis. Studies that do not provide information on the age of participants.

Intervention:

Inclusion: Children undergoing adenoidectomy/A&T and/or children with OSAS/SDB/snoring undergoing A&T. Exclusion: Interventions other than adenoidectomy/T&A or A&T.

Comparison:

Inclusion: Control groups composed of children without ATH, children without OSA, and children without snoring. Comparisons between pre- and post-A&T results. Exclusion: Studies without a direct comparison before and after the intervention or lacking comparison between groups.

Outcome:

Primary and Secondary: Changes in cardiac function, improvements in cardiac dynamics, and assessments of pulmonary blood pressure after adenoidectomy or A&T in children with OSA or ATH. Cardiac alterations in children with OSA compared to controls. Exclusion: Significant comorbidities that could influence cardiac outcomes independently of the presence of ATH or OSA. Unrelated additional interventions (e.g., cardiac surgery) that could independently impact cardiac outcomes. Studies or participants not relevant to the specific population of children with ATH, OSA, or other conditions of interest.

Study Design:

Inclusion: Observational with a control group (observational and comparative), temporal perspective (prospective, observational, cross-sectional, and longitudinal), unspecified temporal structure (cross-sectional, comparative, observational, retrospective studies). Exclusion: Reviews, systematic reviews, meta-analyses, abstracts, and letters.

We applied strict exclusion criteria, which included the removal of articles written in languages other than English, as well as reviews, case reports, letters, studies involving adults (aged > 18 years), studies lacking specific outcome measures, and duplicate studies that had been published multiple times or identified through various data sources.

Two independent reviewers meticulously reviewed the data extraction process for each study, thereby reducing the potential for errors and interpretational biases. In cases where discrepancies arose between the reviewers, a third reviewer was consulted to address these issues, ensuring the accuracy and consistency of data extraction. Additionally, the reviewers evaluated the methodological quality of each study, considering the strength of the study design and the validity of the results. This comprehensive assessment aimed to gauge the overall quality of the scientific evidence presented in the included studies. The PRISMA flowchart illustrates the inclusion criteria, exclusion criteria, and the rigorous methodological approach employed in this study (http://www.prisma-statement.org/PRISMAStatement/FlowDiagram, accessed on 31 August 2023).

### Evaluation of the Risk of Publication Quality Distortion

We thoroughly examined potential sources of overall bias that could influence the findings of these studies. These included selection bias, information bias, confounding bias, detection bias, retrospective bias, attrition bias, and expectation bias.

Additionally, we applied the ROBINS-E tool (Risk Of Bias In Non-randomized Studies of Exposure) as a systematic method for evaluating the bias risk in observational epidemiological studies [13]. We employed assessment tools for evaluating the risk of publication quality distortion as per Mcguinness and Higgins [14] (accessed on 6 October 2023). The questions in these tools meticulously assessed the methods and results of the studies, providing ratings of “High”, “Low”, or “Some Concerns” [15].

The ROBINS-I tool was utilized to evaluate potential bias in estimates of comparative intervention effectiveness, i.e., whether studies had harmful or beneficial effects. This assessment was applied in studies where randomization was not used to allocate individual units or clusters of individuals into different comparison groups [13]. The questions in these tools meticulously assessed the methods and results of the studies, providing ratings of “Serious”, “Moderate”, or “Low”.

## 3. Results

**Figure 1:**
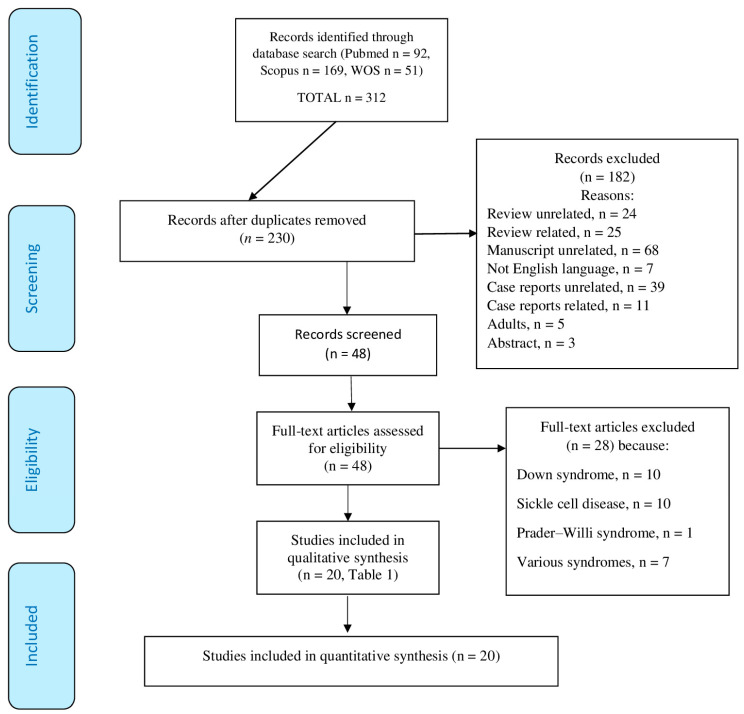
The PRISMA flow diagram visually represents the study selection process and the number of studies included at each stage (31 August 2023).

We initially extracted a total of 230 articles after removing duplicates. After meticulously screening titles and abstracts, we identified 48 studies aligned with our research objectives. Out of these, 28 studies investigated cardiac complications in children with SDB and related syndromes. We then thoroughly examined these studies to assess their relevance and quality further. Following this rigorous evaluation process, we ultimately included 20 articles in our analysis (Figure 1).

Table 1 and Table 2 summarise the cardiac dynamics in individuals with OSA and the results of surgical interventions involving A&T on these physiological factors. The studies incorporated into this compilation offer an in-depth perspective on the clinical implications of such a procedure. Notably, the research presented in the tables has delved into the intricate interaction between ATH, OSA, and cardiological complications within pediatric populations.

Table 1 and Table 2 show studies conducted over the past 20 years, each with a different research purpose and study methods. The available studies can be classified as observational studies (n.2) [16,17], retrospective studies (n.3) [18,19,20], cross-sectional studies (n.4) [21,22,23,24], comparative studies (n.2) [25,26], prospective studies (n.8) [27,28,29,30,31,32,33,34], and clinical trial (n.1) [35].

The total number of children studied was 2429, the total number of cases (children with the condition of interest) was 2172, and the total number of controls (children without the condition of interest) was 257. The minimum number of children in one study was 23 [33], while the maximum number in a study was 620 [19]. The minimum age was 2.5 years [27], and the maximum was 21 years [20]. The authors of these studies used various methods to assess OSA and its effects on the cardiovascular system in children. The diagnosis of OSA was obtained by PSG [18,19,20,21,26,28,35], oximetry [34], and questionnaire OSA-18 [16,17,22,23,32]. Diagnosis of adenoid/tonsillar hypertrophy was obtained using Brodsky’s Scale in three studies [22,23,24] and by taking X-rays in four studies [22,30,32,35]. ENT surgery was reported as adenoidectomy [16,17,20,21,22,25,27,28,29,30,31,32,33,35], tonsillectomy [29,32], and A&T [16,17,20,21,22,25,28,29,30,31,32,33,35]. A cardiac evaluation was performed by Doppler echocardiography [16,17,22,24,27,28,29,30] and the analysis of various cardiac parameters [16,17,25,27,29,30,31,32]. Some studies also involved monitoring biomarkers such as CRP [18] and NT-proBNP [18]. Follow-up was performed in three studies [27,32,33].

The studies presented in Table 2 aim to understand the impact of ATH, OSA, and A&T on the cardiopulmonary health of children, as well as to identify risk factors and the potential benefits of adenoid and tonsil surgery.

### 3.1. Association between ATH-Related Apnea and Cardiac Markers

Some studies examined the potential association between ATH and cardiac markers. Tatlipinar et al. [23] suggested that there may be an association between mean pulmonary artery pressure (mPAP) and specific measures of cardiac function, such as tricuspid annular plane systolic excursion, myocardial performance index of the right ventricle (MPI-RV), and the adenoid-to-nasopharyngeal ratio. Çetin M et al. [30] found that children with ATH had some anomalies in cardiac parameters, such as a thicker interventricular septum and a higher mean pulmonary artery pressure. After surgery, many of these parameters improved and became like the control group. They also observed that pulmonary artery pressure was higher in the preoperative period and improved to average values following A&T.

### 3.2. Prevalence of Pulmonary Hypertension and Associated Risk Factors in Children with OSA

Some studies only investigated the possible association between the severity of OSA and cardiac abnormalities. Attia G et al. [28] highlighted that patients with OSA may exhibit abnormalities in cardiac function related to the severity of OSA and pulmonary pressure. The authors emphasized the importance of cardiac evaluation in children with OSA due to ATH and suggested that surgery can significantly improve cardiac function and pulmonary pressure. Duman D. et al. [16] reported that the first abnormal finding in echocardiography in children with OSA appears to be a significant increase in MPI-RV. Cai XH et al. [26] revealed that children with OSA–hypoventilation syndrome (OSAHS) may exhibit some alterations in cardiac parameters compared to control children and children with primary snoring. The OSAHS group potentially had a higher PH than the control group. However, Clements A.C. et al. [20] concluded that the severity of OSA is not predictive of abnormalities in pre-A&T cardiopulmonary tests. Bitners AC et al. [19] examined the occurrence of PH in a predominantly non-white and urban patient group where the median age was 8.9 years (IQR 5.5–13.1 years), and there was a high prevalence of obesity (72%). The study’s key finding indicates a 4.0% prevalence of elevated RVP among children with severe or very severe OSAS (93.1%) who underwent PH screening. Furthermore, the study did not identify any significant correlation between elevated RVP and clinical or demographic factors, including the severity of OSAS.

Several studies investigated the risk factors for the development of PH. Granzotto E.H. et al. [22] suggested that the ratio between left ventricular ejection time (T) and the pre-ejection period of the A-wave (P) (T/P) could be a valuable indicator to assess systolic pulmonary arterial pressure and identify patients with PH. Omer K.A. et al. [34] reported a low prevalence of PH in children with OSA, and no significant differences were observed in mPAP or other echocardiographic parameters between children with mild–moderate OSA and those with severe OSA. They concluded that PH is rare in children with uncomplicated OSA, and there is no association between PH and the severity of OSA. Marangu D. et al. [24] identified nasal obstruction and a high adenoid-to-nasopharynx ratio as independent risk factors for the development of PH in children with ATH. They reported that approximately one in five children with ATH had PH, with a significant increase in risk when nasal obstruction or a high adenoid-to-nasopharynx ratio was present.

### 3.3. Changes in Biomarkers of Cardiac Stress and OSA

Goldbart AD et al. [21] studied changes in biomarkers of cardiac stress. This study highlighted that OSA was associated with elevated levels of NT-proBNP and inflammation (measured through CRP), suggesting increased cardiac stress. In addition, surgical treatment significantly reduced NT-proBNP levels and improved echocardiographic parameters associated with increased pulmonary pressure in children with OSA.

### 3.4. Effects of A&T on Heart Function

Some of the studies included in this review evaluated the effects of A&T on heart function. Abd El-Moneim E.S. et al. [27] observed an improvement in cardiac dynamics after surgery, with an increased flow through the tricuspid and pulmonary valves, improved RV filling function, and reduced RV size. They suggested that relief from upper airway obstruction through adenoidectomy might lead to improved RV filling, RV output, and a reduction in pulmonary artery pressure. Çetin M et al. [29] highlighted that the surgery positively impacted cardiac function and average pulmonary artery pressure, leading to significant improvements in echocardiographic parameters. After the intervention, these parameters were similar to those of the control group, suggesting a normalization of pulmonary artery pressure. The authors concluded that A&T may positively impact the cardiac function of children with ATH. Cincin A. et al. [17] found that patients with ATH had a higher average pulmonary artery pressure before surgery. After the surgical intervention, a significant improvement in moderate pulmonary artery pressure was observed. The authors suggested that surgery for ATH may significantly affect LV and RV function. Kim D.Y. et al. [31] highlighted that A&T had improved RV function, as evidenced by improved MPI-RV in children with OSA associated with ATH. The intervention did not significantly influence mean pulmonary artery pressure and other cardiac parameters. Bahgat A. et al. [32] observed that the surgery positively affected the patients’ systolic pulmonary artery pressure, leading to normalization within 2 months after the operation. The authors suggested that ATH can cause higher pulmonary artery pressure in children with OSA and that A&T represented an effective therapeutic measure in such patients. Duman D. et al. [16] found that the MPI-RV was initially higher than the control group in patients with ATH, but it improved significantly after A&T to reach values similar to those of the control subjects. They suggested that grade 3 and 4 ATH might increase the MPI-RV, indicating subclinical RV dysfunction, and A&T can reverse these cardiac alterations. Koc S et al. [25] highlighted that A&T significantly improved cardiac function in the study patients. Specifically, there was an improvement in tricuspid valve function, a reduction in the MPI-RV, and a decrease in mean pulmonary artery pressure.

### 3.5. Risk of Bias

From Table 1 and the description of the provided data, it is possible to identify several potential biases in the individual studies. In prospective studies, participant selection could be influenced by the presence of specific symptoms or pre-existing conditions [27,28,30,31,32,33,34]. This could lead to underestimating or overestimating the severity of OSA in participants (selection bias). The lack of detail on the duration of OSA symptoms in some studies could result in a non-representative selection of patients (selection bias) [17,18,19,20,23,25,26,28,29,30,31,32,34,35]. Lack of details on the assessment of the severity of OSA in some studies [24,29,30,33] could lead to variability in data collection and the definition of diagnostic criteria (measurement bias).

The lack of information on follow-up measures in some studies could influence the assessment of outcomes over time (measurement bias). In some studies, the omission of information on age at follow-up, OSA severity index at follow-up, and duration of follow-up could affect the completeness and reliability of the reported data (reporting bias). The absence of detailed follow-up data, such as duration and measures used, could impact the long-term evaluation of OSA severity and its effects (follow-up bias).

The lack of detailed data on follow-up, such as duration [17,18,19,20,22,23,24,34], the SDB measures used, questionnaires [17,22,23,25,27,31,32], OSA-18 score [16,17,22,23,32], PSG [18,19,20,21,26,28,35], and clinical symptom questionnaires [24,29,30,33], could influence the long-term assessment of OSA severity and its effects (follow-up bias). The variation in the patient recruitment period (from January 2008 to September 2023) could affect the results (temporal bias).

**Figure 2:**
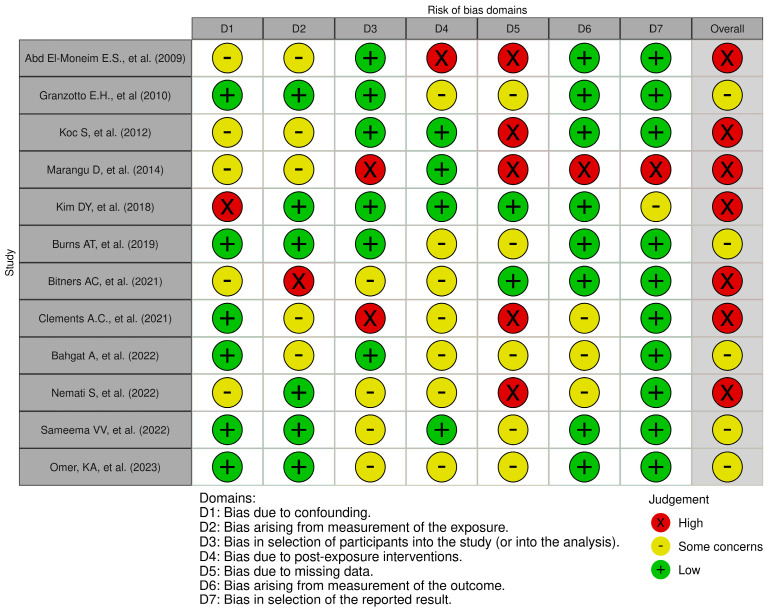
Risk-of-bias plot (ROBINS-E) [18,19,20,22,24,25,27,31,32,33,34,35].

In Figure 2, the results of the risk-of-bias plots with ROBINS-E are presented. Many of the listed studies appear to have significant bias issues, which decreases the reliability of their results.

Figure 2 illustrates the findings regarding three distinct levels of bias, categorized as “Low” risk, “Some concerns”, and “High” risk. The assessment of bias arising from confounding revealed it to be problematic or at high risk in up to 46.2% of the studies. Similarly, bias resulting from exposure measurement was deemed problematic or at high risk in 53.8% of the studies. Participant selection bias was also identified as problematic or at high risk in 53.8% of the studies. Post-exposure intervention bias was found to be problematic or at an increased risk in 61.5% of the studies. Moreover, missing data bias emerged as a significant concern, with a high risk identified in 84.6% of the studies. Lastly, bias related to outcome measurement was considered problematic or at high risk in 30.8% of the studies. In contrast, bias in the selection of reported results was viewed as problematic or at high risk in 23.1% of the studies. In summary, the overall risk of bias was categorized as high or with some concerns in all studies analysed. At least one form of bias that raised significant concerns was identified for every study.

**Figure 3:**
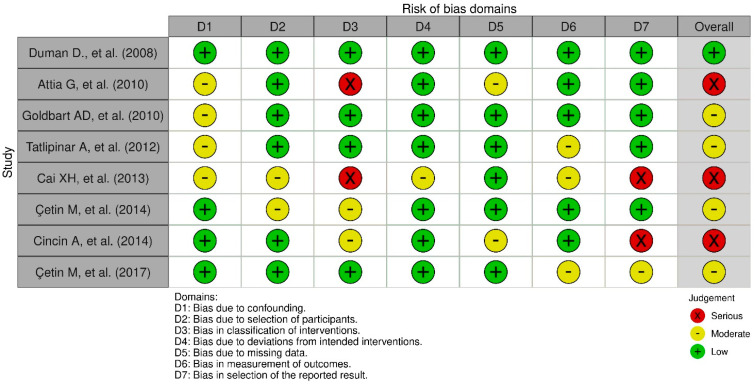
Risk-of-bias plots (ROBINS-I) [16,17,21,23,26,28,29,30].

In Figure 3, the results of the risk-of-bias plots with ROBINS-I are presented. Many of the listed studies appear to have significant bias issues, which raises doubts about the reliability of their results.

Figure 3 displays the findings for three distinct levels of bias, categorized as “Low” risk, “Moderate”, and “Serious” risk. Moderate or severe risk bias due to confounding was observed in 50% of the studies, while participant selection bias was exhibited in 25% of the studies. Deviation from planned interventions was displayed in 12.5% of the studies, and missing data raised concerns in 25%. Both outcome measurement and the selection of reported results were associated with moderate or severe risk bias in 37.5% of the studies.

In summary, an overall moderate or severe risk was found in 87.5% of the studies, indicating that many studies exhibited concerns with moderate or severe bias. Specifically, the analysis underscores that bias due to confounding, participant selection, intervention classification, outcome measurement, and selection of reported results are the primary areas of concern.

## 4. Discussion

This review suggests that ATH and OSA are risk factors for developing cardiac abnormalities, including PH. In addition, there is a potential therapeutic value of A&T in improving cardiac function in children with OSA caused by ATH. However, it is essential to note that the severity of OSA does not serve as a precise predictor for the onset of PH in these patients.

It is essential to acknowledge that this analysis reveals an overall concern regarding the risk of bias assessment, with several cases identified as high risk. It is precisely confounding that missing data presents the most significant concerns. Addressing these challenges is essential to ensure reliable and high-quality outcomes for future research.

OSA has been identified as a cause of severe cardiac complications, including PH and cor pulmonale [36]. The treatment of SDB has been shown to decrease mean pulmonary artery pressure [37]. An examination of 21 studies in 2015 revealed that the management of SDB, primarily through A&T, led to a substantial reduction in multiple cardiovascular parameters [37]. Apneas and hypopneas lead to rapid changes in pleural pressure, hypoxia, and sympathetic nervous system activation, increasing mean pulmonary artery pressure [38]. A systematic literature review of 13 studies (with the latest survey conducted in 2017) reported that A&T appears to improve cardiovascular function in pediatric patients with ATH [39]. It also showed increased LV and RV ejection time and decreased interventricular septum diameter and right ventricular end-diastolic diameter [39].

However, studies on the prevalence and severity of PH in children with SDB are inconsistent. One study reported a relatively high prevalence of PH in echocardiography in a cohort of children with severe OSA [40]. Other recent studies suggest that PH is relatively rare in children with SDB [18,34]. When present, PH has been reported as mild and clinically insignificant by another recent study [34].

Generally, research is based on observational data and may be influenced by inadequately considered confounding factors. Particularly in prospective studies, there are concerns about participant selection, with the possibility of the underestimation or overestimation of OSA severity due to the lack of details regarding symptom duration. Additionally, the lack of information on OSA severity assessment and diagnostic criteria could lead to variability in the results. The absence of detailed follow-up data, including duration and measures used, could affect the long-term evaluation of outcomes and their effects. Variations in the patient recruitment period could also influence results over time. Therefore, it is essential to consider and address these potential biases when interpreting the results.

Pathological modifications observed in PH due to SDB and intermittent hypoxia include hypertrophy of the medial vascular layer and obstructive proliferation of the intima layer in the distal pulmonary arteries [41]. Crucial factors that play a fundamental role in these processes include hypoxic vasoconstriction, mechanical changes arising from overinflated lungs, capillary loss, and inflammation [41]. However, the biological underpinnings of PH in pediatric SDB are still under investigation. Inflammation can damage the vessel walls, making them more prone to constriction and increased pressure [42]. Hypoxia can damage the pulmonary blood vessels, causing them to constrict and increase pressure [42,43]. Endothelial dysfunction induced by OSA [42], specifically, the impairment of the layer of cells lining the inner walls of blood vessels, makes them less capable of regulating blood pressure. Dysregulation mechanisms associated with hypoxic episodes observed in SDB contribute to the onset of PH [41].

The enrolled studies demonstrated an improvement in cardiovascular parameters after the surgical removal of tonsils and adenoids [16,17,20,21,27,28,29,30,31,32]. This result suggests that cardiac involvement is not irreversible at pediatric age and, therefore, it improves in children with OSA caused by ATH following A&T surgery.

The persistence of OSA and SDB after the surgical treatment of tonsils and adenoids can occur in pediatric patients with genetic conditions associated with craniofacial malformations and upper airway abnormalities [44]. Some of the genetic diseases at high risk of persistent OSA are Down syndrome [35,45], Prader–Willi syndrome [46], achondroplasia [47], and other craniofacial syndromes [44,48]. However, despite the high prevalence of SDB in children with Down syndrome, studies on the effects on cardiovascular control are limited [49]. In one study, the priority of cardiological screening in children with Down syndrome or evidence of nocturnal hypoventilation has been addressed [36]. Individuals with Prader–Willi syndrome exhibited compromised cardiac autonomic balance due to reduced parasympathetic modulation during slow-wave sleep. This result may imply an underlying increased cardiovascular risk [50]. Therefore, monitoring these patients closely and considering additional cardiovascular risk management measures is crucial.

This study emphasizes the roles of ATH and OSA as risk factors for cardiovascular complications, especially in pediatric patients with PH. It appears that the first abnormality in echocardiography related to OSA is a significant increase in the MPI-RV [16]. However, the specific timeline of cardiac complications is not clearly defined in the provided texts. A&T is the first-choice treatment for addressing OSA-related cardiovascular complications in otherwise healthy children. The initial finding in echocardiography related to OSA after adenoidectomy is a reduction in PAPm [27]. However, in children with genetic conditions, A&T treatment, when indicated, is often insufficient for complete recovery, making the control of cardiovascular complications more challenging. Therefore, additional therapeutic measures are frequently implemented [51]. Further, well-conducted studies are needed in otherwise healthy ATH patients and fragile children with genetic conditions.

To better address the impact of adenoidectomy and/or tonsillectomy on cardiac parameters in children with upper airway obstruction and OSA, we recommend that future studies prospectively enrol children with confirmed OSA diagnoses, matched for sex and comorbidities, and use standardized methods to evaluate cardiovascular function. Long-term monitoring would enable the evaluation of the effects of surgical and/or medical therapies over time. These studies would provide valuable insights into the prevalence of the cardiovascular effects of OSA in children and the relative effectiveness of different treatments on cardiovascular outcomes.

## 5. Conclusions

OSA can negatively affect cardiac function in pediatric patients and A&T can help alleviate these effects. Research indicates that A&T can positively impact cardiac function in patients with both ATH and OSA. The effectiveness of A&T may vary, which underscores the importance of tailoring clinical decisions to individual circumstances. Recognizing potential complications of OSA in specific patient subgroups and conducting personalized assessments and treatments to optimize outcomes in the care of these patients is essential. Including a comprehensive cardiac evaluation as part of the clinical management for patients with ATH and OSA is highly recommended.

## Figures and Tables

**Table 1:** The table presents various authors’ studies from 2003 to 2023, each with distinct research objectives, subjects, and methodologies.

First Author	Type of Study	Purpose	Cases (Number, Age)	Controls (Number, Age)	Methods	Length of Disease before Diagnosis	OSA Severity Indices at Diagnosis	Age at Follow-up	OSA Severity Index at Follow-up	Follow-up Duration
	**OBSERVATIONAL**									
Duman D. et al. (2008) [16]	Observational study with a control group	MPI-RV in ATH, OSA, and PH. Effects of A&T on MPI.	21 children with grade 3 and 4 ATH (13 males, 7.3 ± 1.8 years).	21 healthy children matched by age and sex (14 males, 7.2 ± 2.2 years).	Doppler echocardiography before and after A&T. OSA-18 questionnaire.	≥6 months (ATH duration not known)	OSA-18 score = 83 ± 27	Not reported	OSA-18 score = 36 ± 12	7.3 ± 2.0 months
Cincin A. et al. (2014) [17]	Observational study	Subclinical cardiac dysfunction in patients with symptoms due to ATH and the echocardiographic impact of A&T.	30 children with grade 3 or 4 ATH (age 7.86 ± 3.83 years)	30 control children, matched for age and sex (age 8 ± 2.77 years)	Echocardiographic and Doppler parameters; tissue Doppler parameters of RV and LV myocardial performance. OSA-18 questionnaire. Repeat echocardiographic examination after A&T.	Not reported	OSA-18 questionnaire	Not reported	Not reported	Not reported
	**RETROSPECTIVE**									
Burns A.T. et al. (2019) [18]	Retrospective analysis	The occurrence of PH in children with OSA and the potential predictors of an elevated PH risk.	163 children (age 7.7 ± 4.8 years), AHI 5.5 events/h (IQR 2.4–12.1 events/h)	Not available	PSG. PH in children is defined as a mean pulmonary arterial pressure ≥ 25 mmHg, right heart catheterization.	Not reported	PSG, AHI 5.5 events/hour, IQR 2.4–12.1 events/hour	Not reported	Not reported	Not reported
Bitners A.C. et al. (2021) [19]	Retrospective review	Prevalence of elevated RV pressure as a marker of PH in children with OSAS.	620 children with OSA, age 8.9 (5.5–13.1) years	Not available	PSG. Echocardiogram: PH screening within 6 months of PSG. Pulmonary vascular resistance elevated above right atrium pressure or elevated pulmonary vascular resistance.	Not reported	PSG (mild, moderate, severe)	Not reported	Not reported	Not reported
Clements A.C. et al. (2021) [20]	Retrospective review	OSA severity level and cardiopulmonary comorbidities that could be identified via preoperative testing.	358 children (age 5.9 ± 3.6 years; range 1.1–21.8 years) with severe OSAS undergoing A&T (genetic syndromes, prematurity, congenital heart disease, and pulmonary comorbidities were included).	Not available	PSG and preoperative testing. oAHI, hypoxia and hypercapnia, severity of OSAS.	Not reported	PSG = 30.3 (23.8)	Not reported	Not reported	Not reported
	**CROSS-SECTIONAL**									
Goldbart AD et al. (2010) [21]	Cross-sectional and longitudinal	Relationship between NT-proBNP and cardiovascular function in children with OSA.	90 children with OSA (age 20 ± 7 months).	45 healthy children.	Children undergoing A&T for OSA, PSG, echocardiography, and CRP and NT-proBNP assay. Three months after A&T, 72 children were re-examined for NT-proBNP and CRP assay.	3 months	AHI 16.9 ± 16 events/hour	Not reported	Not reported	3 months
Granzotto E.H. et al. (2010) [22]	Cross-sectional study	Association of palatine tonsil size (T/P, radiography) and pulmonary artery pressure measured by Doppler echocardiography in children with an indication for A&T.	45 children (age 72.0 ± 32.3 months).	Not available.	Brodsky scale; OSA-18 questionnaire; Palatine tonsil size according to Shintani; Doppler ecocardiogram. Children with an indication for A&T.	24.7 ± 27.8 (2–168) months	OSA-18 = 86.20 ± 20.60 (31–126)	Not reported	Not reported	Not reported
Tatlipinar A et al. (2012) [23]	Cross-sectional study	Association between upper airway obstruction and cardiopulmonary complications.	95 children with OSA and ATH; 4 groups: only hypertrophic adenoids (n. 40, age 6.96 ± 2.11 years); only hypertrophy of the tonsils (n.6, age 7.00 ± 1.54 years); hypertrophic adenoids and tonsils (n.35, 6.69 ± 1.68 years)	14 children (age 7.21 ± 2.08 years)	Brodsky score and adenoids-to-nasopharynx ratio. OSA-18 and Brouilette’s questionnaire. Transthoracic two-dimensional echocardiography.	Not reported	OSA-18, Brouilette classification	Not reported	Not reported	Not reported
Marangu D. et al. (2014) [24]	Cross sectional hospital-based survey	Prevalence and associated PH factors in children with ATH.	123 children aged 2.5 (IQR 1.4–3.5) years with adenoid hypertrophy and OSA	Not available	Brodsky classification and Friedman classification. Doppler echocardiography to determine PH.	Median 14 (IQR 2–51) months	Clinical symptoms	Not reported	Not reported	Not reported
	**COMPARATIVE**									
Koc S. et al. (2012) [25]	Comparative study	RV function and mean pulmonary artery pressure in children with ATH undergoing A&T.	27 children (age 8 ± 2 years) with only ATH.	Not available.	Grades 3 or 4 hypertrophy of the tonsils. A&T and echocardiogram.	Not reported	Brodsky scale	Not reported	Not reported	3 months
Cai X.H. et al. (2013) [26]	Comparative observational study	Relationship between snoring and morbidity in children.	152 snoring children: 63 primary snorers (age 6.02 ± 2.79 years), 89 with OSA (age 5.57 ± 2.55 years)	60 controls (age 6.00 ± 2.48 years)	PSG. Maxillofacial malformations, echocardiogram.	Not reported	AHI = 15.6 events/hour (5.1–85.7)	8.50 ± 2.17 years	Not reported	Approximately 3 years
	**PROSPECTIVE**									
Abd El-Moneim E.S. et al. (2009) [27]	Prospective crossover observational study	Changes in RV performance parameters after adenoidectomy in children with adenoid hypertrophy.	30 children with adenoidal hypertrophy (median age 5 years, range 2.5 and 12 years).	Not available.	Follow-up echocardiographic examination. Brouilette’s questionnaire. Echocardiogram and cardiac Doppler examination one day before and at the follow-up visit.	Duration of obstructive apnea symptoms 2.2 (1.2–9) years	Brouilette score (>3.5)	Not reported	Not reported	36 (30–52) days
Attia G. et al. (2010) [28]	Prospective study	Impact of OSA on myocardial performance using tissue Doppler, echocardiography, and after A&T.	42 children with OSA (5 ± 3.14 years)	45 healthy children matched by age and gender.	PSG (AHI), echocardiography; tissue Doppler ultrasound. A&T, re-evaluated by PSG and echocardiography.	Not specified	PSG, AHI 11.74 ± 2.6 events/hour	Not reported	Not reported	6–8 months
Çetin M. et al. (2014) [29]	[Prospective study]	RV function before and after A&T in children with ATH.	41 children (age 6.0 ± 2.5 years): 15 adenoidectomies, 26 tonsillectomies	40 control children (age 6.0 ± 2.4 years).	Tissue Doppler, pulse echocardiogram, and conventional echocardiography preoperatively and at follow-up.	Not reported	Questionnaire of symptoms	Not specified	Not reported	6 months
Çetin M. et al. (2017) [30]	Prospective study	LV function in children with ATH; effects of A&T on LV function by comparing pre- and post-operative data.	30 children (age 5.9 ± 2.1 years) with upper airway obstruction, who underwent adenoidectomy/T&A.	30 healthy children (age 5.9 ± 2.1 years).	Tissue Doppler echocardiography, conventional echocardiography, before and after A&T. Sinus radiographs and Brodsky scale.	Not reported	Questionnaire	Not reported	Not reported	6 months
Kim D.Y. et al. (2018) [31]	Prospective cohort study	To assess the impact of A&T on RV function in children with OSA caused by ATH.	37 children (7.72 ± 2.22 years) underwent T&A.	Not available	Cohen and Konak method and Brodsky scale, STOP questionnaire, transthoracic echocardiography before and after A&T.	Not reported	STOP Questionare	Not reported	Not reported	12 months
Bahgat A. et al. (2022) [32]	Prospective study	To establish pulmonary arterial systolic pressure in children with OSA with ATH. To evaluate whether A&T has any effect on pulmonary blood pressure.	50 children (age 8.34 ± 3.57 years) with loud snoring and OSA due to ATH.	Not available	Brodsky scale, OSA-18 questionnaire, lateral X-ray of the nasopharynx, echocardiography. A&T: 3 months follow-up after A&T.	Not reported	OSA-18 questionnaire	Not reported	Not reported	3 months
Sameema V.V. et al. (2022) [33]	Prospective study	Parameters of cardiac function via echocardiography before and after A&T in children with ATH.	23 children (age 7.43 ± 2.19 years; range 4–12 years) with ATH.	Not available	Echocardiographic examination prior to A&T surgery. Follow-up with echocardiographic examination.	2.22 ± 1.47 years	Clinical criteria	Not reported	Not reported	3 months
Omer K.A. et al. (2023) [34]	Prospective observational study	Incidence of PH in children with suspected OSA and association between PH and OSA.	170 children (age 3.8 years, IQR 2.7–6.4 years). Children with comorbidities are excluded.	Not available	MOS score: MOS 1–2 (mild-moderate) and MOS 3–4 (severe). PH = mean pressure in the pulmonary artery on echocardiography.	Not reported	Overnight oximetry (McGill Oximetry Score, ODI)	Not reported	Not reported	Not reported
	**CLINICAL TRIAL**									
Nemati S. et al. (2022) [35]	Quasi-experimental clinical trial study	To evaluate the A&T effects on cardiac function in children with snoring and OSA (AHI: 12.2 ± 7.02 events/hour) due to ATH.	42 children (age 7–11 years) with snoring and ATH (grades 3 and 4), A&T candidates.	Not available	Brodsky classification, lateral neck X-ray, PSG. Echocardiography performed one week before and after A&T.	Not reported	PSG, AHI 12.24 ± 7.02 events/hour	Not reported	Not reported	3–6 months

Legend: AHI, apnea–hypopnea index; ATH, adenotonsillar hypertrophy; A&T, adenotonsillectomy; CRP, C-reactive protein; IQR, interquartile range; LV, left ventricle; MPI, myocardial performance index; MPI-RV, myocardial performance index of the right ventricle; NT-proBNP, peptide natriuretic di tipo B; oAHI, obstructive AHI; OSA, obstructive sleep apnea; OSAS, obstructive sleep apnea syndrome; OSA-18, Questionnarie obstructive sleep apnea-18; PH, pulmonary hypertension; PSG, polysomnography; RV, right ventricle; T/P, tonsillar–pharyngeal ratio.

**Table 2:** The table shows the results of studies conducted from 2003 to 2023 in which cardiac outcomes and authors’ conclusions were evaluated.

First Author	Interpretation of Cardiology Findings	Authors’ Conclusions
OBSERVATIONAL		
Duman D. et al. (2008) [16]	MPI-RV initially higher in children with Grade 3 and 4 ATH than controls. MPI-RV improved following A&T similar to the controls.	ATH increases the MPI-RV and subclinical RV dysfunction. A&T can reverse these changes.
Cincin A. et al. (2014) [17]	Before surgery: Patients with symptoms of OSA due to ATH had higher mPAP and impaired RV function. After surgery: Patients with symptoms of OSA from ATH had significant effects on both LV and RV function.	Before surgery patients with ATH showed higher mPAP and after surgery they showed significant improvement.
RETROSPECTIVE		
Burns A.T. et al. (2019) [18]	Low prevalence of PH in pediatric patients with suspected OSA. None of the patients with PH had severe OSA.	PH in pediatric OSA is relatively low.
Bitners A.C. et al. (2021) [19]	High RV pressure was present in a low percentage of children (4%). High RV pressure did not appear related to OSA severity or low oxygen levels during sleep.	Prevalence of elevated RV pressure in children with OSA is low. Severe disease and obesity are risk factors for PH development in children with OSA.
Clements A.C. et al. (2021) [20]	Children with very severe OSA (oAHI ≥ 60 events/hour) underwent more pre-operative cardiopulmonary tests. OSA severity did not predict abnormal findings.	Severity of OSA is not predictive of pre-A&T cardiopulmonary abnormalities.
CROSS-SECTIONAL		
Goldbart A.D. et al. (2010) [21]	OSA was associated with high NT-proBNP levels (increased cardiac stress). Surgical treatment reduced NT-proBNP. Inflammation (increased CRP) was related to alterations in tricuspid flow rate.	NT-proBNP levels increase in children with OSA and decrease following A&T. Echocardiographic parameters suggest an increase in pulmonary pressure in children with OSA that decreases after treatment.
Granzotto E.H. et al. (2009) [22]	The T/P ratio help to assess systolic pulmonary blood pressure and identify patients with PH.	Good correlation between T/P and mPAP in children with ATH and surgical indications for SDB.
Tatlipinar A. et al. (2012) [23]	Correlation between mPAP and cardiac function indicators (including tricuspid annular plane systolic excursion, MPI-RV, and adenoidal–nasopharyngeal ratio).	Patients with ATH are at increased risk of cardiopulmonary complications and associated with more severe OSA symptoms.
Marangu D. et al. (2014) [24]	One fifth of children with ATH had PH. Nasal obstruction (3-fold) and adenoidal-to-nasopharyngeal ratio >0.825 (5-fold) increased the risk.	Nasal blockage and adenoidal hypertrophy are risk factors for PH.
COMPARATIVE		
Koc S. et al. (2012) [25]	A&T led to improvements in cardiac function. Enhancements in tricuspid valve function decreased MPI-RV and reduced mPAP.	A&T improves MPI-RV and reduces mPAP.
Cai X.H. et al. (2013) [26]	Children with OSA and with primary snoring had greater alterations in cardiac parameters than controls.	Children with OSA have higher mPAP.
PROSPECTIVE		
Abd El-Moneim E.S. et al. (2009) [27]	Following adenoidectomy, cardiac dynamics improved: increased flow through the tricuspid and pulmonary valves, improved RV filling function, and reduced RV size.	Relief of OSA by adenoidectomy results in improved RV filling and RV output and reduced mPAP.
Attia G. et al. (2010) [28]	Cardiac function abnormalities in mPAP are related to OSA severity, and are reversible by surgical treatment.	Cardiac evaluation in children with OSA due to ATH is essential. Surgical treatment significantly improves heart function and PH.
Çetin M. et al. (2014) [29]	Surgery positively impacted heart function and mPAP in children with ATH who improved after surgery.	A&T have positive impact on heart function in children with ATH.
Çetin M. et al. (2017) [30]	Children with ATH had abnormalities in cardiac parameters (thicker interventricular septum and a higher mPAP). After surgery, these parameters improved.	mPAP in patients with ATH is higher in the preoperative period and improves following A&T.
Kim D.Y. et al. (2018) [31]	A&T led to an improvement in RV function (improvement in the MPI-RV in children with OSA). Intervention did not significantly affect the mPAP or maximal velocity of tricuspid regurgitation.	OSA from ATH impaired RV function.
Bahgat A. et al. [32]	Surgery positively affected pulmonary arterial systolic pressures, with normalization within 2 months of the operation.	ATH can cause higher pulmonary arterial systolic pressure in children with OSA. A&T is an effective therapeutic measure.
Sameema V.V. et al. (2022) [33]	A&T led to a reduction in mPAP and improved RV function. Diastolic RV dysfunction improved in some patients.	ATH can cause reversible subclinical cardiac dysfunction, which improves after A&T.
Omer K.A. et al. (2023) [34]	Small percentage of children with OSA developed HP. No substantial disparities in mPAP or other parameters between children with mild-to-moderate OSA and severe OSA.	PH is rare in children with uncomplicated OSA. No association between PH and OSA severity. No differences in clinical severity and OSA in children with and without PH.
CLINICAL TRIAL		
Nemati S. et al. (2022) [35]	A&T led to significant improvements in RV function. RV function indices improved after surgery.	A&T improves cardiac function indices in patients with primary snoring, RV function, and reduced pulmonary blood pressure.

Legend: AHI, apnea–hypopnea index; ATH, adenotonsillar hypertrophy; A&T, adenotonsillectomy; CRP, C-reactive protein; BMI, body mass index; IQR, interquartile range; LV, left ventricle; MPI, myocardial performance index; MPI-RV, myocardial performance index of the right ventricle; NT-proBNP, peptide natriuretic di tipo B; OSA, obstructive sleep apnea; OSAHS, obstructive sleep apnea–hypopnea syndrome; OSAS, obstructive sleep apnea syndrome; OSA-18, Questionnarie obstructive sleep apnea-18; mPAP, mean pulmonary artery pressure; PH, pulmonary hypertension; PSG, polysomnography; RV, right ventricle; SDB, sleep-disordered breathing.

## Data Availability

Data sharing is not applicable to this article as no datasets were generated or analyzed during the current study.
